# Evidence for Cytoprotective Effect of Carbon Monoxide Donor in the Development of Acute Esophagitis Leading to Acute Esophageal Epithelium Lesions

**DOI:** 10.3390/cells9051203

**Published:** 2020-05-12

**Authors:** Katarzyna Magierowska, Dominik Bakalarz, Dagmara Wójcik, Edyta Korbut, Aleksandra Danielak, Urszula Głowacka, Robert Pajdo, Grzegorz Buszewicz, Grzegorz Ginter, Marcin Surmiak, Sławomir Kwiecień, Anna Chmura, Marcin Magierowski, Tomasz Brzozowski

**Affiliations:** 1 Department of Physiology, Jagiellonian University Medical College, 31-531 Cracow, Poland; dominik.bakalarz@uj.edu.pl (D.B.); dagmara1.wojcik@uj.edu.pl (D.W.); edyta.korbut@uj.edu.pl (E.K.); aleksandradanielak26@gmail.com (A.D.); urszula.glowacka@uj.edu.pl (U.G.); mppajdo@cyf-kr.edu.pl (R.P.); grzegorz.ginter@uj.edu.pl (G.G.); marcin.surmiak@uj.edu.pl (M.S.); skwiecien@cm-uj.krakow.pl (S.K.); anna.1.chmura@uj.edu.pl (A.C.); m.magierowski@uj.edu.pl (M.M.); 2 Department of Forensic Toxicology, Institute of Forensic Research, 31-033 Cracow, Poland; 3 Department of Forensic Medicine, Medical University of Lublin, 20-090 Lublin, Poland; g.buszewicz@umlub.pl; 4 Department of Internal Medicine, Jagiellonian University Medical College, 31-066 Cracow, Poland

**Keywords:** carbon monoxide, esophageal epithelial cells, reflux esophagitis, gastrointestinal inflammation, interleukin-1 family

## Abstract

Exposure to acidic gastric content due to malfunction of lower esophageal sphincter leads to acute reflux esophagitis (RE) leading to disruption of esophageal epithelial cells. Carbon monoxide (CO) produced by heme oxygenase (HMOX) activity or released from its donor, tricarbonyldichlororuthenium (II) dimer (CORM-2) was reported to protect gastric mucosa against acid-dependent non-steroidal anti-inflammatory drug-induced damage. Thus, we aimed to investigate if CO affects RE-induced esophageal epithelium lesions development. RE induced in Wistar rats by the ligation of a junction between pylorus and forestomach were pretreated i.g. with vehicle CORM-2; RuCl_3_; zinc protoporphyrin IX, or hemin. CORM-2 was combined with NG-nitro-L-arginine (L-NNA), indomethacin, capsazepine, or capsaicin-induced sensory nerve ablation. Esophageal lesion score (ELS), esophageal blood flow (EBF), and mucus production were determined by planimetry, laser flowmetry, histology. Esophageal Nrf-2, HMOXs, COXs, NOSs, TNF-α and its receptor, IL-1 family and IL-1 receptor antagonist (RA), NF-κB, HIF-1α, annexin-A1, suppressor of cytokine signaling (SOCS3), TRPV1, c-Jun, c-Fos mRNA/protein expressions, PGE_2_, 8-hydroxy-deoxyguanozine (8-OHdG) and serum COHb, TGF-β1, TGF-β2, IL-1β, and IL-6 content were assessed by PCR, immunoblotting, immunohistochemistry, gas chromatography, ELISA or Luminex platform. Hemin or CORM-2 alone or combined with L-NNA or indomethacin decreased ELS. Capsazepine or capsaicin-induced denervation reversed CORM-2 effects. COHb blood content, esophageal HMOX-1, Nrf-2, TRPV1 protein, annexin-A1, HIF-1α, IL-1 family, NF-κB, c-Jun, c-Fos, SOCS3 mRNA expressions, and 8-OHdG levels were elevated while PGE_2_ concentration was decreased after RE. CO donor-maintained elevated mucosal TRPV1 protein, HIF-1 α, annexin-A1, IL-1RA, SOCS3 mRNA expression, or TGF-β serum content, decreasing 8-OHdG level, and particular inflammatory markers expression/concentration. CORM-2 and Nrf-2/HMOX-1/CO pathway prevent esophageal mucosa against RE-induced lesions, DNA oxidation, and inflammatory response involving HIF-1α, annexin-A1, SOCS3, IL-1RA, TGF-β-modulated pathways. Esophagoprotective and hyperemic CO effects are in part mediated by afferent sensory neurons and TRPV1 receptors activity with questionable COX/PGE_2_ or NO/NOS systems involvement.

## 1. Introduction

Esophageal mucosa, similar to gastric mucosa is exposed to damaging factors including non-steroidal anti-inflammatory drugs (NSAIDs), ethanol, and many others [1,2,3,4]. However, as it has been clinically observed, the most common pathogenic factor affecting its integrity and physiological structure have internal origin [5,6]. Precisely, within the course of gastroesophageal reflux disease (GERD) and due to the malfunction of the lower esophageal sphincter (LES) esophageal epithelium could be exposed to acidic stomach content, very often mixed with bile leading to the development of esophagitis [7,8]. Some studies reported that refluxed gastric juice-mediated inflammation occurs through the chemokine stimulation and the dysregulation of an immune system-mediated injury rather than corrosive damage and these mechanisms seem to be involved in the development of early histological changes observed in esophagitis [9]. Furthermore, chronic exposure to lower gastrointestinal (GI) tract content leads to the development of GERD symptoms and pathomorphological alterations including premalignant intestinal metaplasia within esophageal epithelium known as Barrett’s esophagus which can progress to esophageal adenocarcinoma (EAC) [10,11,12].

The gaseous molecule, carbon monoxide (CO), next to hydrogen sulfide (H_2_S) and nitric oxide (NO) is involved in the mechanism of gastric mucosal integrity maintenance and gastroprotection via the regulation of gastric blood flow (GBF) and vasorelaxation but whether CO can affect esophageal integrity and esophageal protection still remains unknown [13,14,15,16,17]. CO is produced endogenously via heme degradation by the activity of inducible heme oxygenase 1 (HMOX-1) and constitutive HMOX-2 [14]. CO/HMOX has been shown to mediate anti-inflammatory or anti-oxidative processes in various experimental models of gastrointestinal (GI) pathologies by modulation of many molecular pathways. This includes gastro- and intestinal protective activity of CO donors against NSAIDs-induced gastrotoxicity, oxidative gastric damage, or experimental colitis [13,14,18]. Moreover, the CO-releasing pharmacological donor, tricarbonyldichlororuthenium (II) dimer (CORM-2) has been shown to accelerate the healing of chronic gastric ulcers mediated by the activation of various cellular pathways and alteration of growth factors expression and release, independently on the endogenous H_2_S production [19]. CO released from its pharmacological donor has been shown to affect duodenal secretion of HCO_3_^−^ [20]. Interestingly, anti-oxidative HMOX was shown to be involved in the regulation of VIP-induced LES relaxation [21].

The possible esophagoprotective activity of CO-releasing prodrugs has not been explained so far. Therefore, our present study was designed to investigate for the first time if endogenous CO produced by the activity of HMOX-1/Nuclear factor (erythroid-derived 2)-like 2 (Nrf-2), and/or intragastric (i.g.) administration of CORM-2 affects esophageal mucosa exposed to experimental acute reflux esophagitis (RE) and accompanying alterations in esophageal blood flow (EBF). We assessed the possible involvement of afferent sensory nerve activity, endogenous NO, prostaglandins (PG) biosynthesis pathways with special emphasis on esophagoprotective PGE_2_ produced by cyclooxygenases (COXs), and the alterations in cellular hypoxia and DNA oxidation within esophageal mucosa pretreated or not with CORM-2. Furthermore, we analyzed the changes in mRNA/protein expression of anti/proinflammatory markers and selected components of complex inflammatory response pathways in serum and in esophageal mucosa of rats pretreated with CO donor and exposed to experimental RE.

## 2. Materials and Methods

### 2.1. Experimental Design, Animals, Chemicals, and Drugs

Eighty Wistar rats, with average weight 250–300 g were fasted for 24 h before the experiments with free access to drinking water. All procedures were approved by the I Local Ethical Committee for Care and Use of Experimental Animals, held by Faculty of Pharmacy, Jagiellonian University Medical College in Cracow (Decision No.: 176/2018; Date: 26 September 2018). Experiments were run with implications for replacement, refinement, or reduction (the 3Rs) principle and in compliance with the ARRIVE guidelines.

All chemicals and drugs used for experiments and molecular or biochemical assessments were purchased from Sigma-Aldrich (Schnelldorf, Germany) unless otherwise stated. All compounds were administered i.g. using orogastric tube or injected intraperitoneally (i.p.) as reported previously [19].

Animals were randomized to the appropriate experimental groups and treated i.g. with (1) DMSO and water (1:10) as a solvent for the administered compounds (further called: vehicle) or (2) CO releasing CORM-2 [19,22], applied in gradually increased doses: 0.5, 1, 2.5, or 5 mg/kg (series A). In separate series B, rats with RE were pretreated i.g. with: (3) hemin, HMOX-1 inducer [23], applied in a dose of 5 mg/kg previously reported to accelerate gastric ulcer healing [24], (4) zinc protoporphyrin IX (ZnPP), the HMOXs inhibitor [25], administered in a dose of 10 mg/kg, previously reported in our studies to decrease CO content in gastric mucosa [26], (5) RuCl_3_ as a negative control to CORM-2 without ability to release CO [24], applied in a dose of 2.5 mg/kg corresponding to the most effective esophagoprotective dose of CORM-2 selected in this study. Additional groups of rats were pretreated with CORM-2 applied in a dose of 2.5 mg/kg i.g., selected in experiments of series A, administered alone or in combination with (6) N^G^-nitro-L-arginine (L-NNA, 20 mg/kg i.p.), non-selective NO synthase (NOS) inhibitor [27], (7) indomethacin (5 mg/kg i.p.), non-selective COX inhibitor [24], or (8) capsazepine (5 mg/kg i.g.) as the synthetic antagonist of TRPV1 receptors [28]. In a separate group of animals (series C), capsaicin was administered for three days in a dose of 25, 50, and 50 mg/kg (total dose: 125 mg/kg s.c.) two weeks before the start of the study, to induce the functional ablation of afferent sensory nerve as described previously [29]. In one of the subgroups of series C, these rats with capsaicin-denervation were pretreated with CORM-2 (2.5 mg/kg i.g.).

Acute esophageal lesions due to RE were induced 30 min after each pretreatment has been completed, as described previously [30]. Briefly, under isoflurane anesthesia (Aerrane, Polska Grupa Farmaceutyczna, Lodz, Poland), the abdomen was opened, the pylorus and the transitional junction between the forestomach and the corpus were exposed and ligated with silk suture to induce RE [30]. Next, the abdomen was closed contemporarily and all RE rats remained at the conscious state for 4 h.

### 2.2. Determination of EBF Level, Macroscopic and Microscopic Assessment of Esophageal Lesion Score and Biological Samples Collection

After 4 h of RE, under isoflurane anesthesia, the EBF was determined by laser flowmetry as described previously [24]. Briefly, the EBF was measured in esophageal mucosa not involving RE lesions using laser flowmeter (Laserflo, model BPM 403A, Blood Perfusion Monitor, Vasamedics, St. Paul, Minnesota, USA). Average values of three measurements for each rat were determined and expressed as a percent of the value obtained in esophageal mucosa of intact rats.

After measurement of EBF, blood was collected from vena cava and whole blood or serum samples were stored in −20 °C for further analyses. Immediately after blood has been collected, rats were sacrificed by i.p. administration of a lethal dose of pentobarbital (Biowet, Pulawy, Poland), the esophagus was excised, opened longitudinally, rinsed with saline, and the macroscopic esophageal lesion score (ELS) was determined by planimetry.

The lesion score was calculated and expressed based on macroscopic and microscopic degree of injury 0–5 according to following criteria:

0—no visible lesions,

1—hemorrhagic dot-like erosions covering up to 50% of esophageal mucosa (observed up to 2 cm proximally from gastroesophageal junction (GEJ)) with leukocytes infiltration,

2—hemorrhagic dot-like erosions covering more than 50% of esophageal mucosa (observed up to 4 cm proximally from GEJ) with leukocytes infiltration,

3—extensive hemorrhagic linear and dot-like erosions covering up to 50% of mucosal surface (reaching the length up to 2 cm proximally from GEJ) with leukocytes infiltration and extensive epithelium desquamation,

4—extensive hemorrhagic linear and dot-like erosions covering 50–75% of mucosal surface (reaching the length up to 4 cm proximally from GEJ) with leukocytes infiltration and extensive desquamation of epithelium,

5—extensive hemorrhagic linear erosions covering more than 75% of mucosal surface (reaching the length up to 4 cm proximally from GEJ) with leukocytes infiltration and extensive desquamation of epithelium.

Esophageal mucosal samples were scraped off on ice, snap-frozen in liquid nitrogen, and stored at −80 °C until further biochemical and molecular analyses [24].

### 2.3. Esophageal Lesions Histology, Mucus Production, and Selected Proteins Distribution Assessment Preparation

For histology, esophageal tissue sections were excised and fixed in 10% buffered formalin, pH 7.4. Samples were dehydrated by passing them through a series of alcohols with incremental concentrations, equilibrated in xylene for 10–15 min and embedded in paraffin; paraffin blocks were cut into about 4 μm sections using a microtome. The prepared specimens were stained with haematoxylin/eosin (H/E) or alcian blue/periodic acid-Schiff (AB/PAS) [31].

To evaluate gastric mucosal Nrf-2 and HMOX-1 proteins distribution within esophageal mucosa immunohistochemistry was employed as described previously [31]. Briefly, endogenous peroxidase was blocked by 3% H_2_O_2_ solution in abovementioned specimens and incubated in a water bath in the presence of EDTA or sodium citrate and treated with proteinase K. Next, tissue slides were stained with anti-HMOX-1 mouse monoclonal antibody (1:500; 66743-1-Ig; Proteintech, Manchester, The United Kingdom) and Nrf-2 rabbit polyclonal antibody (1:200; 16396-1-AP; Proteintech). BrightVision plus Poly-HRP-Anti MS/Rb IgG system containing HRP-linked secondary antibody was further implemented. DAB Quanto (TA-125-QHDX; Thermo Fisher Scientific, Waltham, MA, USA) was used to visualize investigated proteins in esophageal mucosal tissue sections.

Samples were evaluated using a light microscope (AxioVert A1, Carl Zeiss, Oberkochen, Germany) [31]. Digital documentation of histological slides was obtained using above mentioned microscope equipped with automatic scanning table and ZEN Pro 2.3 software (Carl Zeiss, Oberkochen, Germany) to collect multiple photographs of each histological sample and to stitch them into one picture; to obtain a better quality of each picture, the background was subtracted and unified as white [31]. All histological documentation was collected using the same microscopic magnification (20×) and calibrated scale-bars were added digitally and adjusted automatically when photographs were adapted to the same size for the final figure.

### 2.4. Determination of Esophageal mRNA Expression for Selected Genes by Real-Time PCR

Expression of mRNA for HMOX-1, HMOX-2, COX-1, COX-2, iNOS, nNOS, hypoxia-inducible factor (HIF)-1α, annexin-A1, IL-6 receptor (IL-6R), IL-1β, IL-1 receptor 1 and 2 (IL-1R1 and IL-1R2, respectively), IL-1R antagonist (IL-1RA), TNF-α, TNF receptor 2 (TNF-R2), nuclear factor κB (NF-κB), c-Fos, c-Jun, suppressor of cytokine signaling (SOCS3) were determined in esophageal mucosa by real-time PCR, as described previously [31]. Total RNA was isolated using a commercially available kit with spin columns (GeneMATRIX Universal RNA Purification Kit, EURx, Gdansk, Poland) according to manufacturer’s protocol. Reversed trascription to cDNA was performed using High-Capacity cDNA Reverse Transcription Kit (MultiScribe™, Applied Biosystems, Life Technologies, Carlsbad, CA, USA).

RNA concentration was measured using Qubit 4 Fluorometer (Thermo Fisher Scientific, Waltham, MA, USA). RT was normalized for each reaction regarding total RNA concentration to obtain the same value (2 μg) for each sample. Results obtained for RNA samples isolated from healthy (intact) esophageal mucosa and transcribed to cDNA were further used as reference control.

Expression for HMOX-1, HMOX-2, COX-1, COX-2, iNOS, nNOS, HIF-1α, annexin-A1, IL-1β, TNF-α, β-actin (ACTB) and succinate dehydrogenase complex (SDHA) was determined using specific primers [31,32,33]. Expression for IL-6R, IL-1R1, IL-1R2, IL-1RA, TNF-R2, NF-κB, c-Fos, c-Jun, SOCS3 was determined using specific primers described elsewhere [34]. ACTB and SDHA were considered as reference genes. To increase the result quality, all genes of interest were normalized to both housekeeping genes. All samples were assessed for each gene within the same microplate.

Real-time PCR was conducted using thermal cycler Quant Studio 3 (Thermo Fisher Scientific, Waltham, MA, USA) and SYBR Green dye including kit (SG qPCR Master Mix (2x), EURx, Gdansk, Poland). Results were analyzed using ΔΔCt method [32,35]. Results with at least two-fold up- or downregulation of mRNA expression vs. vehicle group and with *p* < 0.05 were considered and marked as statistically and biologically significant.

### 2.5. Determination of Proteins Expression in Esophageal Mucosa by Western Blot

Protein expression for Nrf-2, HMOX-1, HMOX-2, COX-1, COX-2, and TRPV1 in esophageal mucosa was determined using Western Blot, as described previously [32,33]. Rabbit monoclonal anti-HMOX-1 (ab68477, Abcam, Cambridge, UK) in dilution of 1:1000, rabbit polyclonal anti-COX-1 (13393-1-AP, Proteintech, Manchester, UK) in dilution of 1:1000, rabbit polyclonal anti-COX-2 (ab 15191, Abcam) in dilution of 1:1000, rabbit polyclonal anti-HMOX-2 (14817-1AP, Proteintech), rabbit polyclonal anti-Nrf-2 (16396-I-AP, Proteintech) in dilution of 1:500 and mouse monoclonal anti-GAPDH (60004-1-Ig, Proteintech) in dilution of 1:2000 were used as primary antibodies. Protein expression was visualized using horseradish peroxidase-linked secondary anti-rabbit IgG antibody (7074, Cell Signaling Technology) or anti-mouse IgG antibody (7076, Cell Signaling Technology) in dilution of 1:2000 where appropriate. All primary and secondary antibodies were diluted in 5% non-fat milk. Only anti-Nrf-2 antibody was diluted in 5% bovine serum albumin.

Chemiluminescence was developed using WesternSure^®^ ECL Substrate (LI-COR, Lincoln, NE, USA) or WesternBright Quantum (Advansta, Menlo Park, CA, USA) and was measured using C-DiGit^®^Blot Scanner (LI-COR). The intensity of bands was determined and analyzed using Image Studio 4.0 software (LI-COR). The expression of each protein of interest was determined using 5 samples per experimental group and obtained values were normalized to the expression of ACTB as loading control [32,33].

### 2.6. Measurement of COHb Content in Blood Samples by Gas Chromatography (GC)

COHb levels in whole blood samples were determined as reported previously [31]. Briefly, 9.6 mL of water was added to the 400 μL of whole blood samples and homogenized by sonication (Bandelin Sonoplus, Germany). The volume of 2.5 mL of homogenate was pipetted into two 10 mL headspace vials (2 test samples). To obtain calibration samples, about 5 mL of the remaining volume of the homogenate was saturated with CO for 20 min (100% saturation CO). The CO used to saturate the calibration samples was obtained by reacting concentrated sulfuric acid with 80% formic acid. The unbound CO was removed by flushing with nitrogen for 3 min. Calibration solutions with CO saturation 1.25, 2.5, 5, and 10% were prepared from 100% saturated homogenates. The volume of 2.5 mL of each calibration solution was pipetted into headspace vials (4 calibration samples). The vials were then sealed with an aluminum cap and silicon/teflon septum. Each vial was gently flushed with helium for 30 s and then 1.5 mL of 20% potassium hexacyanoferridate was added with a syringe.

For the GC/O-FID-headspace analysis, a Thermo Trace GC Ultra (Thermo Electron Corp., Waltham, MA, USA) equipped with O-FID detector (FID with jet nickel microcatalytic methanizer) was used. The jet nickel microcatalyzer converts CO to methane at 330 °C, which increases the sensitivity of CO detection. The system was equipped with a Thermo TriPlus HS autosampler. The prepared samples were mixed and incubated at 70 °C for 8 min in autosampler agitator to achieve complete CO liberation. The gas-phase of each sample (200 μL) were injected with an autosampler gas-tight syringe (heated at 72 °C). The split/splitess injector (200 °C) with closed split was used. The GC separation was performed with HP-Molesieve column (Agilent Technologies, USA) 30 m/0.53 mm ID/0.25 μm at constant flow 15 mL/min of helium as a carrier gas. The temperature program consisted of the following steps: 60 °C for 2 min followed by 120 °C for 2 min achieved by a heating rate 60 °C/min.

### 2.7. Determination of PGE_2_ and 8-hydroxy-deoxyguanozine (8-OHdG) Concentration in Esophageal Mucosa 

The esophageal mucosal levels of both PGE_2_ and 8-OHdG were assessed as described previously [31]. All samples were assessed within the same assay microplates. PGE_2_ concentration in esophageal biopsies was determined using PGE_2_ ELISA kit (ab133021, Abcam) according to manufacturer’s protocol. Homogenization process of each sample was standardized regarding sample weight and buffer volume and results were expressed in pg/mL of esophageal tissue homogenate. The 8-OHdG content as DNA oxidation marker was assessed in DNA isolated from esophageal mucosa using ELISA kit (589320, Cayman Chemical, Ann Arbor, MI, USA) according to manufacturer’s protocol.

### 2.8. Assessment of Serum Content of Inflammatory Markers by Luminex Microbeads Fluorescent Assays

Determination of TGF-β1, TGF-β2, TGF-β3, IL-1β, IL-6 concentrations in serum was performed using Luminex micro beads fluorescent assays (Bio-Rad, Hercules, CA, USA) and Luminex MAGPIX System (Luminex Corp., Austin, TX, USA). All samples were assessed within the same assay microplates. Results were calculated from calibration curves and expressed in pg/mL, according to the manufacturer’s protocol, as described previously [31].

### 2.9. Statistical Analysis

Experiments and data collection were done by operators blinded to the sample identity. Results were analyzed using GraphPad Prism 5.0 software (GraphPad Software Inc., La Jolla, CA, USA). Results are presented as mean ± SEM and as median with range for esophageal lesion evaluation. Statistical analysis was conducted using Student’s t-test or ANOVA with Dunnett’s multiple comparison post hoc test if more than two experimental groups were compared. Kruskal–Wallis or Mann–Whitney U test were employed appropriately for ordered statistics. The size for each experimental group was of *n* = 5–6. *p* < 0.05 was considered to be statistically significant.

## 3. Results

### 3.1. The Effect of Pretreatment with CORM-2 on Esophageal Lesions, EBF, and Mucus Secretion and Protein Distribution of Nrf-2, HMOX-1 within Esophageal Mucosa

**Figure 1:**
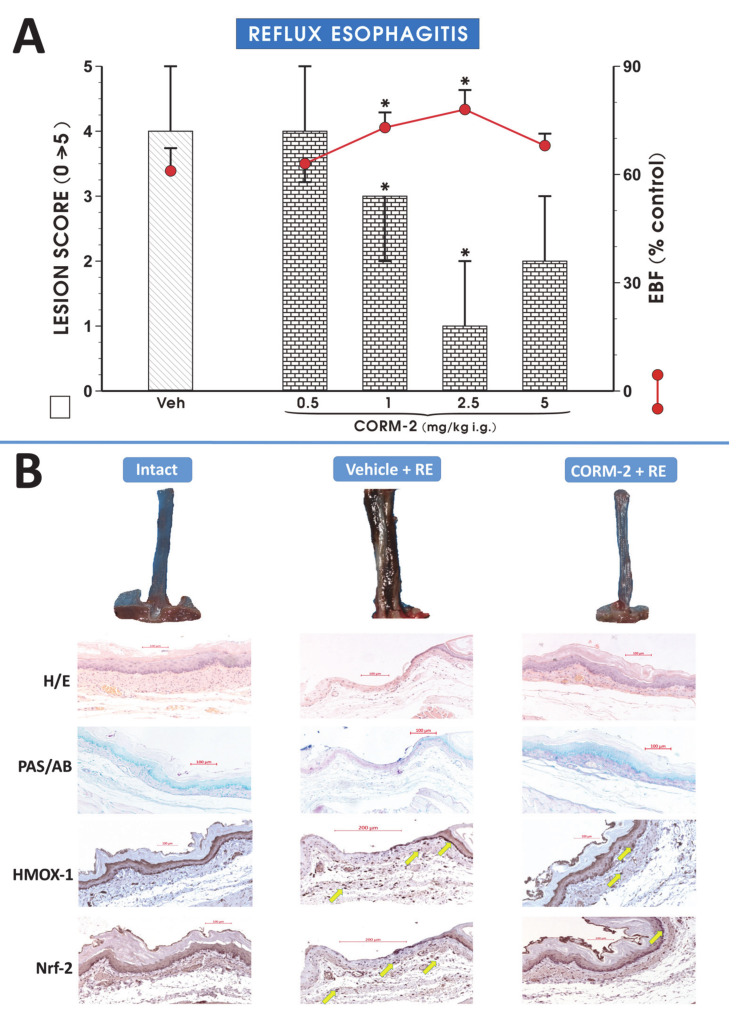
Lesion score (bar chart) and the esophageal blood flow (EBF) (red dots) (**A**) in esophageal mucosa of rats pretreated with vehicle or tricarbonyldichlororuthenium (II) dimer (CORM-2, 0.5–5 mg/kg i.g.) and exposed to 4 h of reflux esophagitis (RE). Intact refers to healthy esophageal mucosa not exposed to RE. Lesion score results are expressed as medians with range of 5 rats per each experimental group. EBF results are mean ± SEM of 5 samples per each experimental group. Significant change as compared with the respective values in vehicle-control group is indicated by asterisk (*p* < 0.05). (**B**) Macroscopic appearance of randomly selected representative esophageal mucosa (excised and opened longitudinally esophagus) exposed or not to RE (upper panel) and randomly selected representative histological slides of esophageal mucosa exposed or not to RE with or without vehicle or CORM-2 pretreatment and stained with H/E, PAS/AB, or anti-HMOX-1 or anti-Nrf-2 antibodies pointed representatively by yellow arrows (lower panel) as a supplement to the data included on Figure 1A and 2C,E,F. All histological photographs were collected under the same magnification (20×) and calibrated scale-bar was digitally added.

Figure 1A shows that i.g. pretreatment with CORM-2 applied in a dose of 1 or 2.5 mg/kg significantly decreased esophageal lesion score and significantly increased EBF as compared with vehicle (*p* < 0.05). Figure 1B shows the macroscopic and microscopic appearance of representative intact esophageal mucosa and RE-induced lesions in rats pretreated i.g. with vehicle or CORM-2 (2.5 mg/kg). In contrast with physiological morphology of intact esophageal mucosa, the extensive acute hemorrhagic erosions covering more than 75% of mucosal surface and reaching the length of more than 2 cm proximally from the gastroesophageal junction (GEJ) were observed macroscopically in esophageal mucosa exposed to RE (Intact vs. Vehicle + RE, Figure 1B). In animals pretreated with CORM-2 (2.5 mg/kg i.g.), the exposure to RE induced acute hemorrhagic dot-like erosions, covering up to 25% of esophageal mucosa and observed up to 2 cm proximally from GEJ (Vehicle vs. CORM-2, Figure 1B).

In rats pretreated i.g. with vehicle but not with CORM-2 (2.5 mg/kg), the exposure of esophageal mucosa to RE resulted in necrotic erosions with almost complete extensive desquamation of epithelium surface, deeply penetrating into lamina propria and accompanied by significant leukocytes infiltration into submucosal layer (Figure 1B, H/E). The mucus layer usually present at the surface of esophageal mucosa of intact rats was completely absent in RE rats pretreated with vehicle but not with CORM-2 as determined by AB/PAS staining (Figure 1B (PAS/AB)). These alterations were accompanied by the increased yield of HMOX-1 and Nrf-2 proteins expression in lamina propria and in the submucosal layer of esophageal wall in RE rats pretreated with vehicle or CORM-2 (Figure 1B (HMOX-1 and Nrf-2)).

### 3.2. Alterations in Esophageal mRNA and/or Protein Expression of HMOX-1, HMOX-2 and Nrf-2 and in Blood Level of COHb in Rats with RE Pretreated with Vehicle or CORM-2

**Figure 2:**
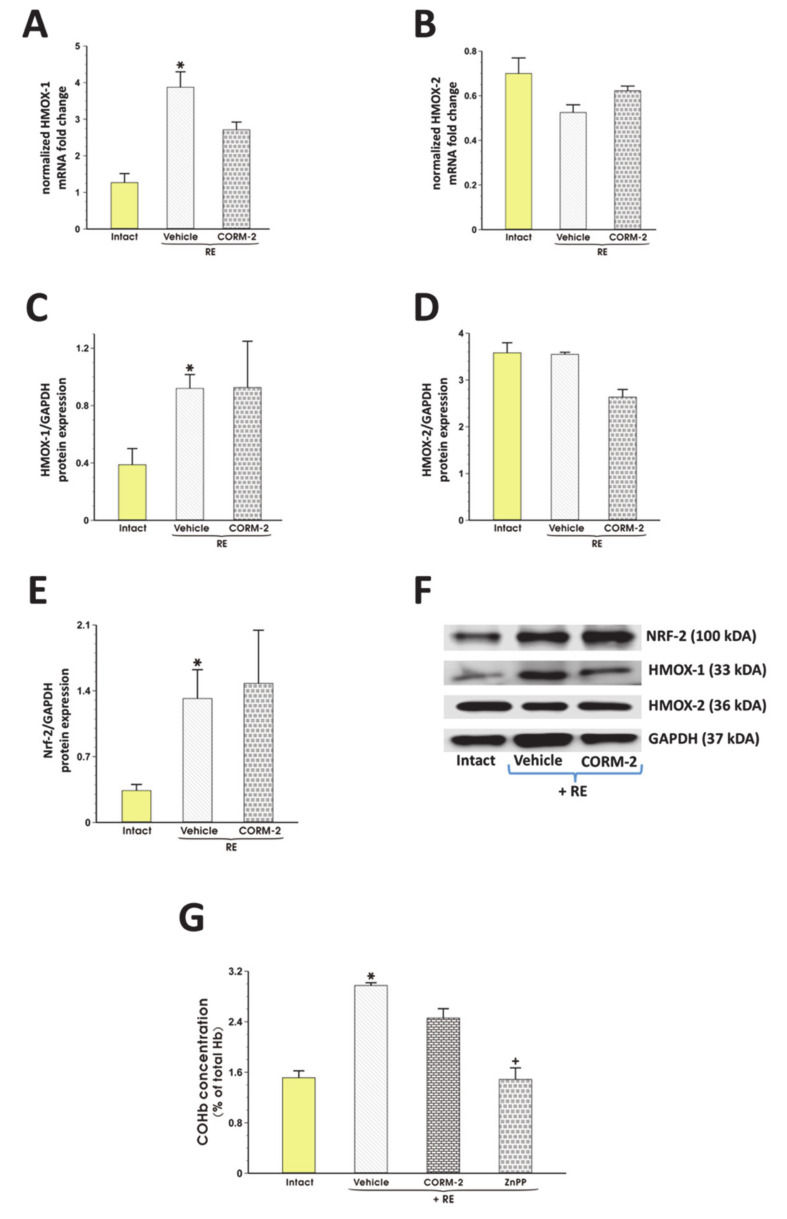
The changes in the expression of HMOX-1 (**A**), HMOX-2 (**B**) mRNA, HMOX-1 (**C**, **F**), HMOX-2 (**D**, **F**), Nrf-2 (**E**, **F**) protein in the esophageal mucosa and the content of carboxyhemoglobin (COHb) (**G**) in blood of rats pretreated i.g. with vehicle or tricarbonyldichlororuthenium (II) dimer (CORM-2, 2.5 mg/kg) and exposed to 4 h of reflux esophagitis (RE). Intact refers to healthy rats not exposed to RE. Results are mean ± SEM of 5 samples per each experimental group. F: The representative esophageal protein expression bands for Nrf-2, HMOX-1, HMOX-2, and ACTB obtained in each experimental group. G: The blood COHb content expressed as a percent of total Hb. ZnPP refers to the group pretreated i.g. with zinc protoporphyrin IX (10 mg/kg). Asterisk indicates a significant change as compared with respective values obtained in intact esophageal mucosa, not exposed to RE (*p* < 0.05). Cross indicates a significant change as compared with the values obtained in the vehicle-pretreated group exposed to RE (*p* < 0.05).

In RE rats pretreated with vehicle, the esophageal mRNA and/or protein expression for HMOX-1 and Nrf-2 but not for HMOX-2 was significantly upregulated as compared with intact rats (*p* < 0.05, Figure 2A–F). Pretreatment with CORM-2 (2.5 mg/kg i.g.) did not significantly affect mRNA and/or protein esophageal expression for HMOX-1, HMOX-2 and Nrf-2 as compared with vehicle (*p* < 0.05, Figure 2A–F). Figure 2G shows that COHb content in blood of rats exposed to RE and pretreated i.g. with vehicle was significantly elevated as compared with intact (*p* < 0.05). Administration of ZnPP (10 mg/kg i.g.) but not CORM-2 significantly decreased COHb concentration in rats with RE-induced erosions as compared with vehicle (*p* < 0.05, Figure 2G).

### 3.3. Involvement of Endogenous NO/NOS, PG/COX, TRPV1 and Afferent Sensory Neurons in CORM-2 Mediated Esophagoprotection and Upregulation of EBF

**Figure 3:**
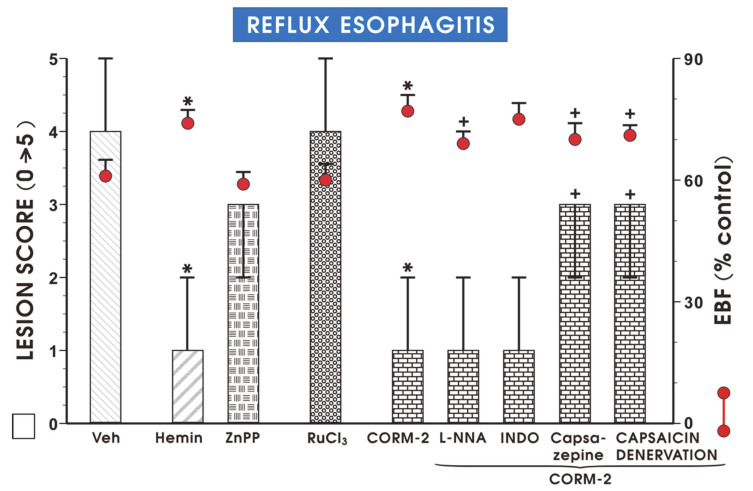
Lesion score (bar chart) and the esophageal blood flow (EBF) (red dots) in esophageal mucosa of rats pretreated i.g. with vehicle, hemin (5 mg/kg), zinc protoporphyrin IX (ZnPP, 10 mg/kg), RuCl_3_ (2.5 mg/kg) or tricarbonyldichlororuthenium (II) dimer (CORM-2, 2.5 mg/kg) administered alone or combined with N^G^-nitro-L-arginine (L-NNA, 20 mg/kg i.p.), indomethacin (INDO, 5 mg/kg i.p.), capsazepine (5 mg/kg i.g.) or capsaicin denervation and exposed to 4 h of reflux esophagitis. Lesion score results are medians with range of 5 rats per each experimental group. EBF results are mean ± SEM of 5 rats per each experimental group. Significant change as compared with the respective values in vehicle-control group is indicated by asterisk (*p* < 0.05). Cross indicates significant change as compared with the group pretreated with CORM-2 alone (*p* < 0.05).

Figure 3 shows that the pretreatment with hemin (5 mg/kg i.g.) or CORM-2 (2.5 mg/kg) but not with ZnPP (10 mg/kg) or RuCl_3_ (2.5 mg/kg) significantly decreased the esophageal lesion score and increased EBF as compared with vehicle (*p* < 0.05). Pretreatment with CORM-2 combined with L-NNA (20 mg/kg i.p.) or indomethacin (5 mg/kg i.p.) did not significantly affect lesion score but L-NNA significantly reduced EBF as compared with CORM-2 administered alone (*p* < 0.05, Figure 3). Pretreatment with CORM-2 with or without the combination with capsazepine (5 mg/kg i.g.) or with capsaicin-denervation significant increase in lesion score and downregulation of EBF was observed as compared with rats with intact sensory nerves administered with CORM-2 alone (*p* < 0.05, Figure 3).

**Figure 4:**
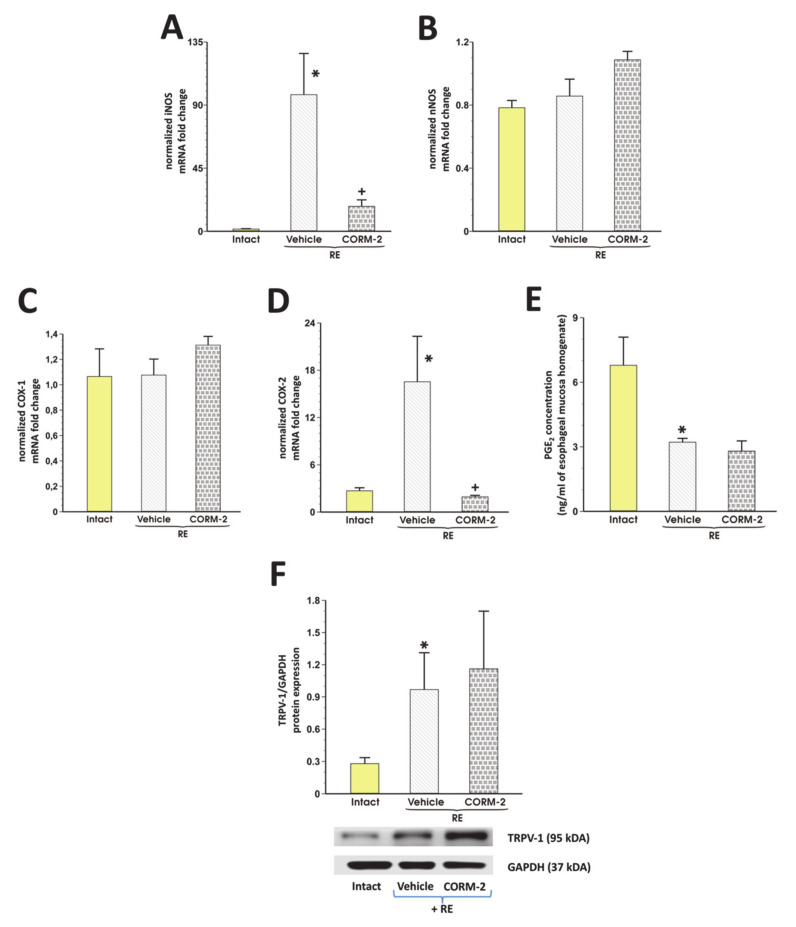
Esophageal expression of iNOS (**A**), nNOS (**B**), COX-1 (**C**), COX-2 (**D**) mRNA, TRPV1 protein (**F**), and PGE_2_ concentration (**E**) in esophageal mucosa of rats pretreated i.g. with vehicle or tricarbonyldichlororuthenium (II) dimer (CORM-2, 2.5 mg/kg) and exposed to 4 h of reflux esophagitis (RE). Intact refers to healthy esophageal mucosa not exposed to RE. Results are mean ±SEM of 5 rats per each experimental group. Results are expressed as fold change of normalized esophageal mucosal iNOS and nNOS mRNA expression. Asterisk indicates a significant change as compared with respective values obtained in intact esophageal mucosa, not exposed to RE (*p* < 0.05). Cross indicates a significant change as compared with the values obtained in the vehicle-pretreated group exposed to RE (*p* < 0.05).

In rats exposed to RE and pretreated with vehicle, esophageal mRNA expression for iNOS and COX-2 but not for nNOS and COX-1 was significantly upregulated as compared with intact rats (*p* < 0.05, Figure 4A–D). Pretreatment with CORM-2 (2.5 mg/kg i.g.) significantly downregulated esophageal mRNA expression of iNOS and COX-2 but not that of nNOS or COX-1 as compared with vehicle (*p* < 0.05, Figure 4A–D). Figure 4e shows that PGE_2_ content in esophageal mucosa of rats exposed to RE and pretreated i.g. with vehicle was significantly decreased as compared with intact (*p* < 0.05). CORM-2 did not significantly affect PGE_2_ concentration in rats with RE-induced lesions as compared with vehicle (*p* < 0.05, Figure 4E). In rats exposed to RE and pretreated with vehicle, esophageal protein expression for TRPV1 was significantly upregulated as compared with intact rats (*p* < 0.05, Figure 4F). Pretreatment with CORM-2 (2.5 mg/kg i.g.) did not significantly affect esophageal protein expression of TRPV1 as compared with vehicle-pretreated RE rats (*p* < 0.05, Figure 4F).

### 3.4. The Effect of CORM-2 on Hypoxia and Oxidation Observed in Esophageal Mucosa with RE

**Figure 5:**
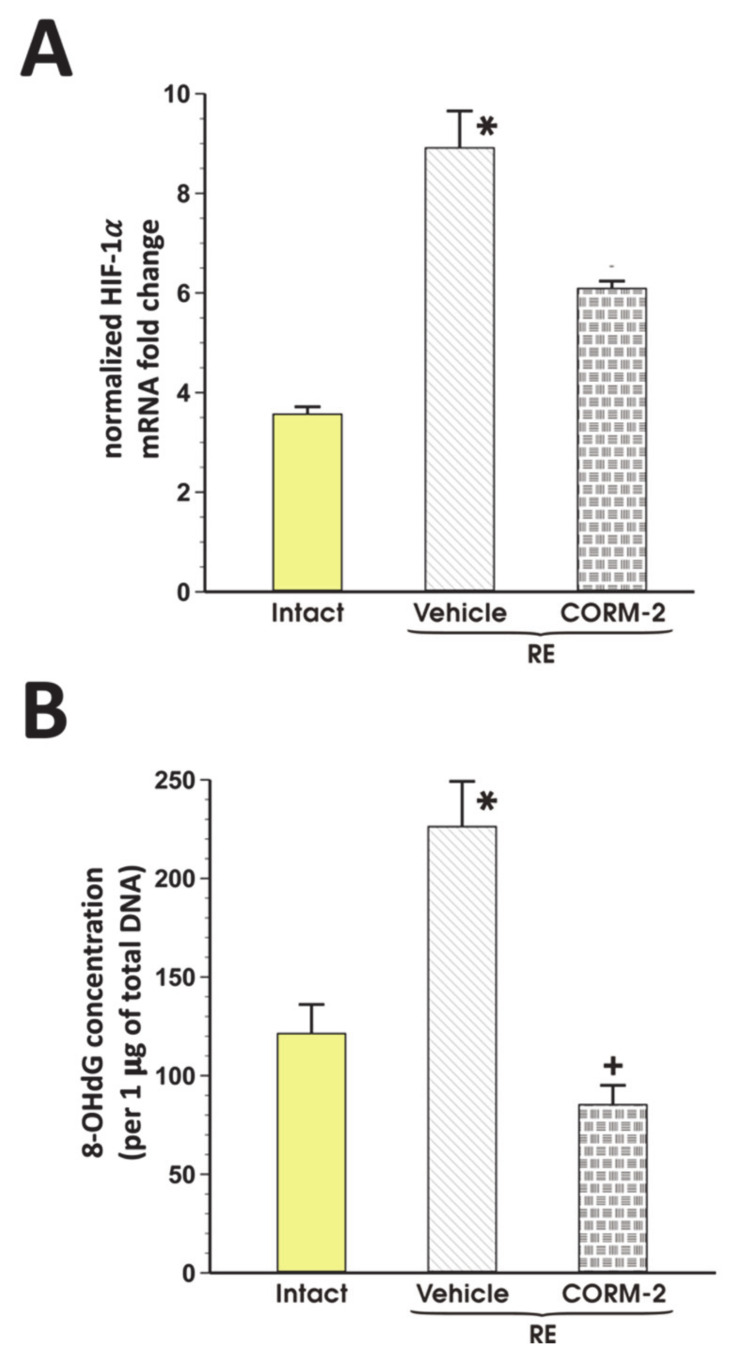
Expression of HIF-1α mRNA (**A**) and 8-hydroxy-deoxyguanosine (8-OHdG) (**B**) content in esophageal mucosa of rats pretreated i.g. with vehicle or tricarbonyldichlororuthenium (II) dimer (CORM-2, 2.5 mg/kg) and exposed to 4 h of reflux esophagitis (RE). Intact refers to healthy esophageal mucosa not exposed to RE. Results are mean ± SEM of 5 rats per each experimental group. Significant change as compared with the respective values in intact group is indicated by asterisk (*p* < 0.05). Cross indicates significant change as compared with the group treated with vehicle (*p* < 0.05).

Figure 5A,B show that in rats exposed to RE and pretreated with vehicle, esophageal mRNA expression for HIF-1α and 8-OHdG concentration was significantly increased as compared with intact rats (*p* < 0.05). Pretreatment with CORM-2 (2.5 mg/kg i.g.) reduced though not significantly the esophageal mRNA expression of HIF-1α and significantly decreased 8-OHdG content as compared with vehicle (*p* < 0.05, Figure 5A).

### 3.5. CORM-2-Mediated Modulation of Systemic and Local Inflammatory Response in Rats Exposed to RE

**Figure 6:**
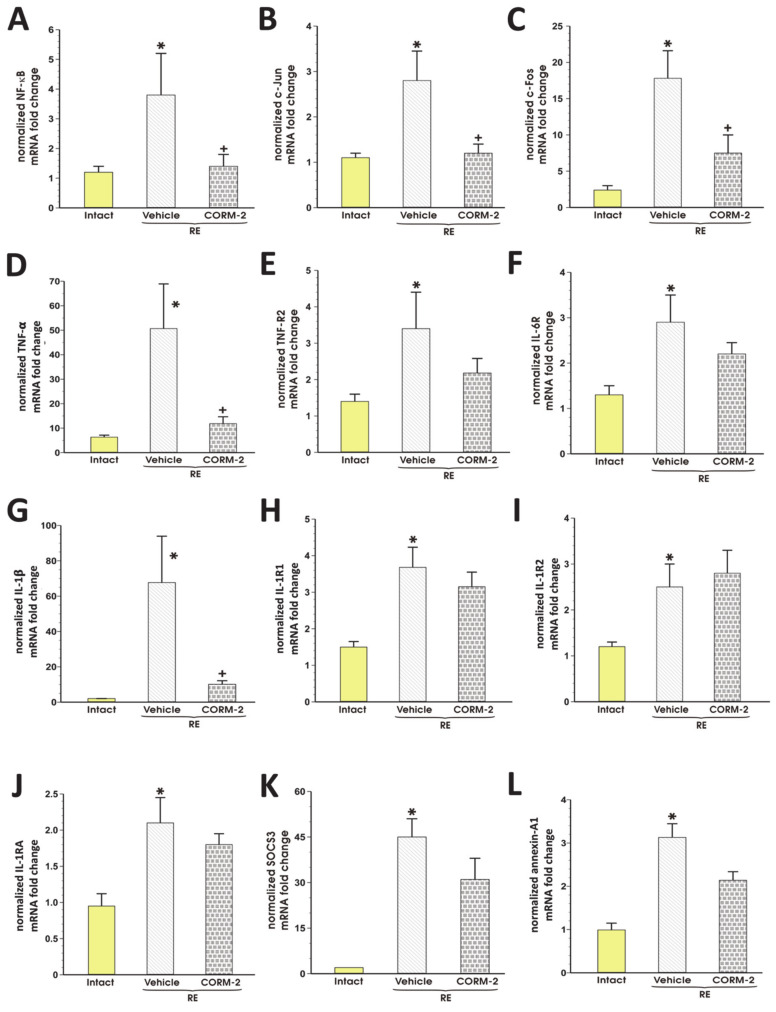
Expression of mRNA for NF-κB (**A**), c-Jun (**B**), c-Fos (**C**), TNF-α (**D**), TNF-R2 (**E**), IL-6R (**F**), IL-1β (**G**), IL-1R1 (**H**), IL-1R2 (**I**), IL-1RA (**J**), SOCS3 (**K**) and annexin-A1 (**L**) in esophageal mucosa of rats pretreated i.g. with vehicle or tricarbonyldichlororuthenium (II) dimer (CORM-2, 2.5 mg/kg) and exposed to 4 h of reflux esophagitis (RE). Intact refers to healthy esophageal mucosa not exposed to RE. Results are mean ±SEM of 5 samples per group. Results are expressed as fold change of normalized esophageal mucosal mRNA expressions. Asterisk indicates a significant change as compared with respective values obtained in intact esophageal mucosa, not exposed to RE (*p* < 0.05). Cross indicates a significant change as compared with the values obtained in the vehicle-pretreated group exposed to RE (*p* < 0.05).

Figure 6A–L shows that in rats exposed to RE and pretreated with vehicle, esophageal mRNA expression for NF-κB, c-Jun, c-Fos, TNF-α, TNF-R2, IL-6R, IL-1β, IL-1R1, IL-1R2, IL-1RA, SOCS3, and annexin-A1 was significantly upregulated as compared with intact rats (*p* < 0.05). Pretreatment with CORM-2 (2.5 mg/kg i.g.) significantly decreased esophageal mRNA expression of NF-κB (A), c-Jun (B), c-Fos (C), TNF-α (D), IL-1β (G) but not of TNF-R2 (E), IL-6R (F), IL-1R1 (H), IL-1R2 (I), IL-1RA (J), SOCS3 (K) and annexin-A1 (L) as compared with vehicle (*p* < 0.05, Figure 6).

**Figure 7:**
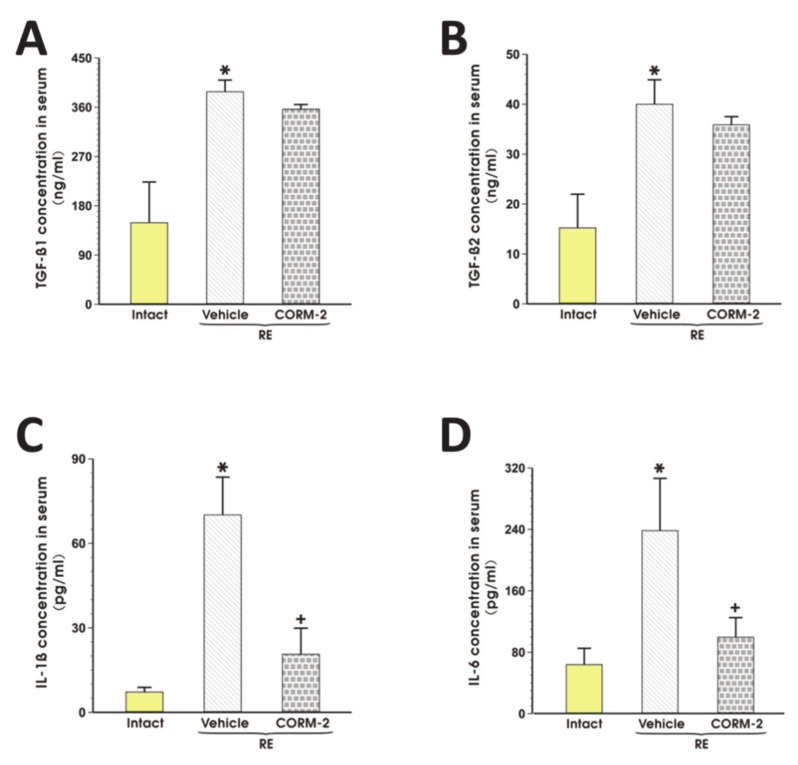
Serum concentration of transforming growth factor (TGF)-β1 (**A**), TGF-β2 (**B**), IL-1β (**C**) and IL-6 (**D**) in rats pretreated i.g. with vehicle or tricarbonyldichlororuthenium (II) dimer (CORM-2, 2.5 mg/kg) and exposed to 4 h of reflux esophagitis (RE). Intact refers to healthy animals not exposed to RE. Results are mean ± SEM of 5 samples per each experimental group. Asterisk indicates a significant change as compared with intact rats (*p* < 0.05). Cross indicates a significant change as compared with the values obtained in the vehicle-pretreated group exposed to RE (*p* < 0.05).

Figure 7A–D shows that the serum concentration of TGF-β1, TGF-β2, IL-1β, and IL-6 in rats exposed to RE and pretreated with vehicle was significantly elevated as compared with intact rats (*p* < 0.05). Pretreatment with CORM-2 (2.5 mg/kg i.g.) significantly decreased serum content of IL-1β and IL-6 but failed to affect the serum content of TGF-β1 and TGF-β2 as compared with respective values of these cytokines in vehicle-pretreated rats (*p* < 0.05, Figure 7A–D).

## 4. Discussion

Esophageal mucosa, similarly to gastric mucosa, is constantly exposed to potentially noxious agents administered orally, including non-steroidal anti-inflammatory drugs (NSAIDs), ethanol, and many other noxious factors [1,2,3,4]. However, squamous esophageal epithelium could be additionally compromised by bile and acidic stomach content influx due to malfunction of lower esophageal sphincter observed in the course of GERD [1,2,3,4,36]. Chronic GERD may lead to the development of premalignant intestinal metaplasia called Barrett’s esophagus with the presence of goblet cells, which are not normally observed within esophageal mucosa [1,2,3,4,36,37,38,39]. This phenomenon could be considered as a defensive esophageal adaptation to the altered physiological conditions [26,27,28,29,30,31,32,33,34,35,36,37,38,39]. However, even single and short-lasting exposure of esophageal structure to the low pH refluxate is known to induce local irritation, oxidation, and inflammation of esophageal mucosa, frequently resulting in necrotic erosions and desquamation of esophageal epithelium described as reflux esophagitis (RE) [27,40,41]. Indeed, in our study employing appropriate experimental animal model described before [30], we observed that RE-induced esophageal lesions were accompanied by the decrease in EBF followed by hypoxia leading to DNA oxidation and increased COHb content. Moreover, we have noticed the enhanced systemic and local inflammatory response. In our study, exposure to RE upregulated esophageal mRNA and/or protein expression of various molecular pathways including annexin-A1, HMOX-1/Nrf-2, NF-κB, IL-1 family members, SOCS3, TRPV1, and many others.

In this study, we have provided for the first time evidence that pretreatment with CO-releasing CORM-2 dose-dependently protected the esophageal mucosa against the formation of macroscopic and microscopic esophageal lesions. This esophagoprotective effect of this CO donor was accompanied by the increase in EBF. Similar preventive and vasodilatory effects were observed after induction of endogenous CO producing HMOX by pretreatment with hemin. This is in pair with previously published data on CO-releasing CORMs in the gut showing the dose-dependent gastroprotective and ulcer healing properties of these CO-releasing tools against acute gastric lesions and chronic acetic acid-induced gastric ulcers [14,15,16,23,42,43]. Precisely, it has been reported that CORM-2 elevated CO content in GI mucosa and exerted its preventive or therapeutic effects due to the modulation of particular molecular pathways in parallel with the increasing concentrations of CO released from its donor when CORM-2 was orally administered [44]. CORM-2 has been also reported to dose-dependently modulate expression of particular molecular factors in vitro including NF-κB [45]. However, it is worth to mention that when high concentration of CORM-2 (i.e., > 50 mg/kg) was introduced, this CO-donor exerted cytotoxic effect within gastric mucosa but interestingly, only slightly increased COHb blood concentration has been observed, below the values considered as toxic in an animal model of ethanol-induced damage [44]. Thus, based on previously published evidence, we have employed the dose range of 0.5 up to 5 mg/kg i.g. of CORM-2 in our study to verify possible beneficial but not toxic effects of CO within esophageal mucosa. Additionally, vasodilatory effects of CO have been observed within the cardiovascular system and within the GI tract [23,26,46,47]. To further confirm that the esophagoprotective activity of CORM-2 is related to CO release but not to the presence of Ru in the chemical structure of this compound, we have documented in this study that pretreatment with RuCl_3_ does not exert the similar effects comparable to that presented by this CO donor. This clearly supports our conclusion that the beneficial effect of CORM-2 against RE-induced esophageal damage could be due to its ability to release CO. Interestingly, we found that mRNA and/or protein expression for HMOX-1 and Nrf-2 are upregulated in esophageal mucosa. Moreover, an increased COHb content in blood of rats exposed to RE was observed and the pharmacological inhibition of HMOX activity decreased COHb concentration but failed to affect RE-induced lesions. Taken together, this suggests that Nrf-2/HMOX-1/CO pathway is activated as a self-defensive feedback mechanism possibly resulting from the esophageal injury which helps to counteract the damaging effects of RE within esophageal mucosa.

The PGE_2_ produced by the enzymatic activity of COX and vasoactive NO are very important components of the gastric mucosal barrier and their inhibition impaired the gastroprotection-induced by various compounds [48]. Our data have shown that pharmacological inhibition of COX-1/COX-2 by indomethacin and NOS by L-NNA, did not affect esophagoprotective activity of CORM-2 suggesting that endogenous PG or NO are not the primary mediators of esophageal protection mediated by CO-donor. Interestingly, L-NNA but not indomethacin reduced hyperemic effect of CORM-2 showing that the impairment of esophageal microcirculation by the NO-synthase inhibition per se is not detrimental for the esophagoprotective activity exhibited by this CO-donor. On the other hand, the CO donor itself decreased esophageal mRNA expression of proinflammatory markers COX-2 and iNOS upregulated in RE rats and failed to affect PGE_2_ concentration decreased in esophageal mucosa compromised by acidic content. Thus, we assume that CO released from CORM-2, at least in this experimental model, acts independently on COX/PGE_2_ and NO/NOS pathways. However, the possibility that endogenous NO may contribute to the increase in the EBF associated with CORM-2-induced esophagoprotection, cannot be excluded. In agreement with this notion, we have reported that CORM-2 attenuated the aspirin-induced gastric damage and the inhibition of NO synthase by L-NNA only partially reduced CORM-2-induced protection, but did not reverse completely this effect [49]. Moreover, the CO donor prevented gastric mucosa against oxidative ischemia/reperfusion injury without involvement of COXs activity [28].

Previous studies reported that afferent sensory fibers releasing the vasodilatory neurotransmitters and TRPV1 receptors are involved in the maintenance of gastric mucosal integrity, gastroprotection, and the regulation of gastric microcirculation [26]. Interestingly, the acid-evoked hyperemia in the esophageal and duodenal mucosa is inhibited by the TRPV1 antagonist capsazepine or sensory denervation, suggesting that acid can activate TRPV1 on sensory nerve fibers [50]. Moreover, the gastroesophageal acid reflux can stimulate esophageal vagal sensory afferents by activating the proton-sensitive TRPV1 channels in rats with experimental RE [51]. In our study, the esophagoprotective and hyperemic effects of CORM-2 were reduced by the ablation of sensory nerves by a high dose of capsaicin or blockade of TRPV1 by capsazepine. Moreover, pretreatment with this CO donor maintained protein expression of TRPV1 upregulated in esophageal mucosa exposed to RE. Therefore, we conclude that afferent sensory nerves activity and TRPV1 are involved in esophageal protection and modulation of EBF by CO.

As described above, exposure to acute reflux leads to the development of severe esophagitis. We observed that epithelial mRNA expression and/or serum levels of inflammatory markers and inflammatory response complex elements interacting together, such as NF-κB, IL-1β, and its receptors, endogenous IL-1RA, TNF-α and its receptor, IL-6 and its receptor, TGF-β1, TGF-β2, annexin-A1, SOCS3, c-Fos, c-Jun, which all were increased in rats with RE. Proinflammatory cytokines, IL-1ß, IL-6 and TNF-α are key players in signaling pathways such as NF-κB activation and mitogen activated protein kinase (MAPK) cascade initiation and appear to play important roles in esophageal diseases including RE [52]. Increased expression of TNF-α and its receptor have been demonstrated in the progression of Barrett’s metaplasia to adenocarcinoma [53]. The elevated TNF-α mRNA has been confirmed in a rat model of chronic acid reflux esophagitis [54]. Zhang et al. reported that inhibition of p38 MAPK pathway in an animal model of reflux-induced esophagitis reduced mRNA levels of IL-1β, IL-6 and TNF-α in esophageal mucosa and protein levels of TNF-α, IL-6, and IL-1β in serum [55]. Additionally, activation of the NF-κB pathway and NF-κB target genes such as: IL-1β, IL-6, and IL-8 in the esophageal epithelium exposed to acid or bile acid has been already implicated [56,57]. IL-1RA or its agonists, IL-1α and IL-1β are the members of the interleukin-1 family. IL-1RA is a naturally occurring competitive inhibitor of IL-1 through binding to its main receptor IL-1R1 [58]. In contrast to IL-1α and IL-1β which promotes inflammation, IL-1RA has been demonstrated to suppress angiogenesis accompanying gastric and esophageal cancer cell growth [59,60]. On the other hand, SOCS proteins are negative regulators of cytokine signal transduction affecting e.g., IL-6 signaling [61]. According to study by a Zafra et al., SOCS proteins may contribute to the development of eosinophilic esophagitis [62]. In their studies, SOCS1 and SOCS3 mRNA levels were significantly increased in esophageal biopsies collected from eosinophilic esophagitis patients [62]. Moreover, c-Fos and c-Jun proteins have been recognized to form the inducible transcriptional complex AP-1 which binds to the TPA-responsive element (TRE)/AP-1 DNA motifs and related sequences [63]. This pathway could be stimulated by TGF-β to modulate cell proliferation [64]. In our study, pretreatment with CO-releasing CORM-2 decreased inflammatory response as manifested by the decreased esophageal mRNA expression for IL-1β, TNF-α, c-Fos, c-Jun, NF-κB. Interestingly, this CO donor maintained increased serum content and esophageal mRNA expression of anti-inflammatory TGF-β1, TGF-β2 and annexin-A1, SOCS3, IL-1RA, respectively, possibly diminishing the effect of still upregulated expression of TNF-R2, IL-6R, IL-1R1, and IL-1R2. Taken together, this novel finding suggests that increased bioavailability of this gaseous molecule could attenuate the inflammatory response to RE on the systemic level and alleviate it locally within esophageal epithelium by the modulation of the abovementioned complex mechanism.

Moreover, the administration of CO donor in RE rats prevented hypoxia maintaining upregulated mRNA expression for HIF-1α and prevented DNA oxidation by the decrease in concentration of 8-OHdG content. Thus, we assume that esophagoprotective effect of CORM-2-induced esophagoprotection may involve the anti-inflammatory and anti-oxidative activity of CO. Indeed, previously published data have shown that HMOX/CO system and CO donors can modulate inflammatory and anti-oxidative response within the digestive system and prevented the GI mucosa against gastric or colonic damage [14,15,16,28,49]. Moreover, it has been reported that HMOX-1 derived metabolites including CO activated the mitochondrial function of astrocytes via HIF-1α/estrogen-related receptor α (ERRα) circuit and therefore play an important role in the repair of neurovascular function after ischemic brain injury [65]. Additionally, CO has been suggested as a protective factor limiting the profibrotic effects of TGF-β in the vasculature [66]. The components of HMOX-1/CO pathway have been shown in hepatocellular carcinoma cells to confer their resistance to TGF-β-mediated growth inhibition by increasing Smad3 phosphorylation via the ERK1/2 pathway [67].

**Figure 8:**
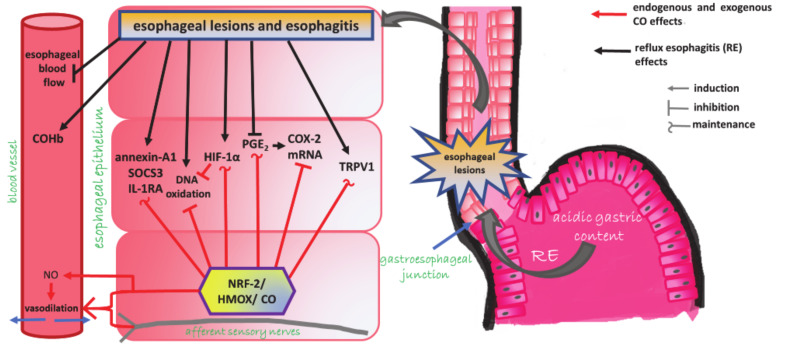
Summary of esophagoprotective effects of carbon monoxide (CO) in animal model of reflux esophagitis.

Taken together, we conclude that CO produced endogenously by Nrf-2/HMOX or released from CORM-2 protects the esophageal mucosa against the damage induced by RE. Esophagoprotective and hyperemic effects of CO are independent on the PGE_2_/COX system, partly dependent on endogenous NO production and may involve the afferent sensory nerves and TRPV1 receptors activity. CO donor prevented DNA oxidation maintaining upregulated mRNA expression for HIF-1α and modulated complex inflammatory response due to the maintenance of elevated anti-inflammatory annexin-A1, SOCS3, IL-RA mRNA esophageal expression, and blood content of TGF-β in rats with RE (Figure 8).
